# Tracing woody-organic tsunami deposits of the 2011 Tohoku-oki event in Misawa (Japan)

**DOI:** 10.1038/s41598-021-88199-3

**Published:** 2021-04-26

**Authors:** Piero Bellanova, Mike Frenken, Yuichi Nishimura, Jan Schwarzbauer, Klaus Reicherter

**Affiliations:** 1 Institute for Neotectonics and Natural Hazards, RWTH Aachen, Lochnerstrasse 4-20, 52056 Aachen, Germany; 2 Institute for Geology and Geochemistry of Petroleum and Coal, RWTH Aachen University, Lochnerstrasse 4-20, 52056 Aachen, Germany; 3 Institute of Seismology and Volcanology, Hokkaido University, Kita-10, Nishi-8, Kita-ku, Sapporo, 060-0810 Japan

**Keywords:** Natural hazards, Environmental chemistry

## Abstract

With a minimum of three reported waves, the 2011 Tohoku-oki tsunami’s destructive force caused massive damage along the northern Japanese Aomori coast. At Misawa the coastal control area was inundated up to 550 m inland and sandy sediment remnants can be traced to c. 350 m (c. 61–63% of the maximum inundation) from the shoreline. Linking the discovery of floatable plastic objects within a woody and organic layer to our analytical data lead to the detection of a yet undocumented woody-organic tsunami deposit first appearing on top of the sandy deposit but then reaching even further inland (approx. 69–72% of the max. inundation). By this observation our understanding of the documented part of the tsunami inundation may be improved. As a consequence, sand sheets of historic and paleo-tsunamis represent minimum estimates for the coastal inundation and underestimation may be reduced by addressing the woody and organic fraction of a tsunami’s inundation.

## Introduction

**Figure 1:**
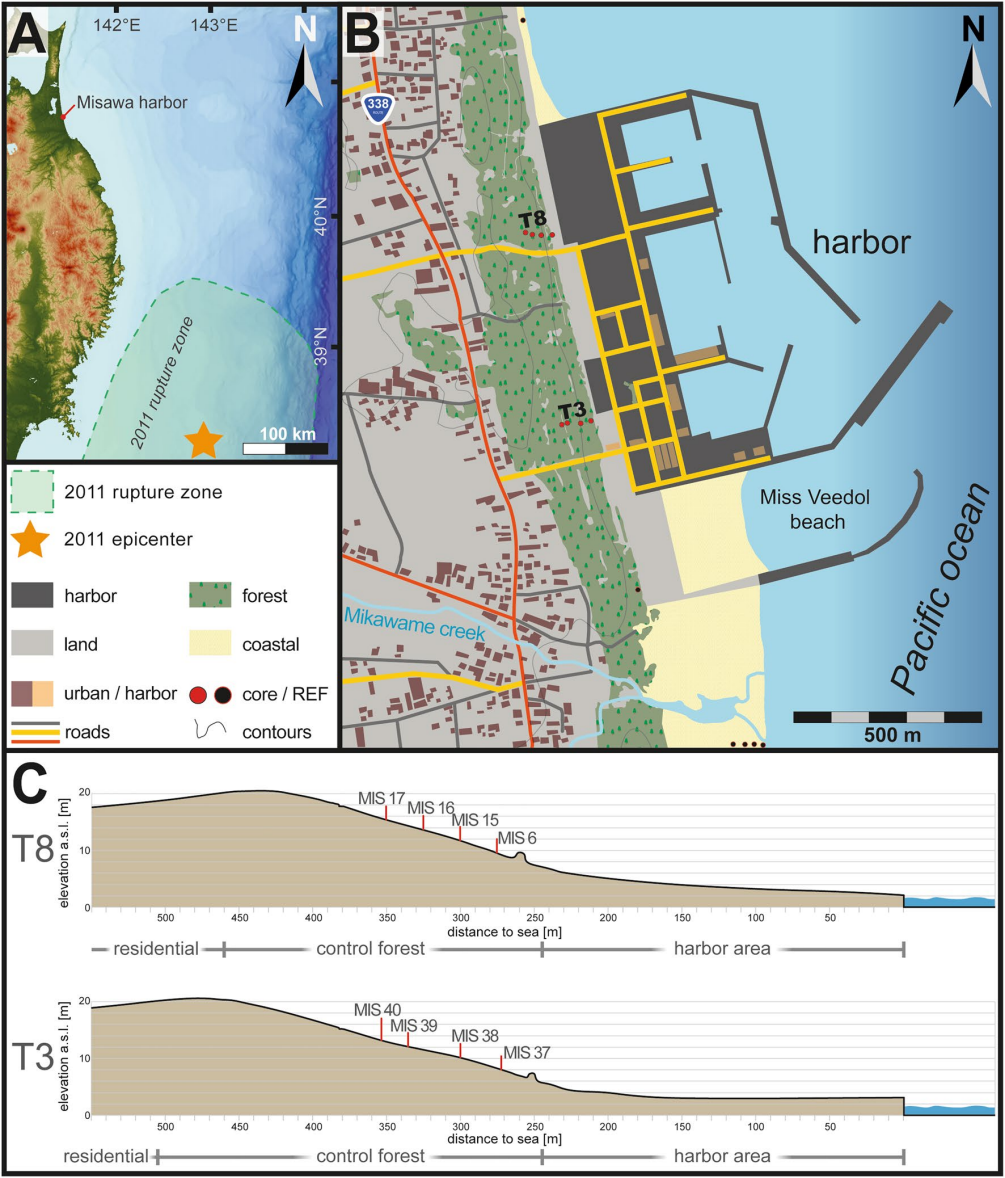
(**A**) Location of the study area in reference to the 2011 Tohoku-oki earthquake rupture zone^41^ and epicenter^42^. (**B**) Study side of the Misawa harbor with the sample locations of transects T3 and T8 labelled by red dots and reference samples with black dots (**C**) Topographical profiles based on SRTM data for transects T3 and T8 with the location of each sampled Geoslicer. Maps and topographic profiles are based on open-access SRTM data (USGS EROS data through EarthExplorer; http://earthexplorer.usgs.gov/), processed using ArcGIS Desktop 10.2 (ESRI) and illustrated Adobe Illustrator (Creative Cloud version 2020, https://adobe.com/products/illustrator).

In the past century, coastal areas around the Pacific Ocean have experienced the highest risk of tsunamis worldwide (e.g., Aleutians 1946; Chile 1960, 2010; Alaska 1964; South Pacific 2009, Japan 2011). Contrasting to many of these tsunami-prone regions, the Japanese islands are densely populated, especially the main island’s economically important low-lying and flat coastal areas^1^. The northeastern coastline (e.g., Aomori, Iwate, Miyagi and Fukushima prefectures; Fig. 1) is in range of the Japan trench with high seismic activity causing at least four events within the nineteenth and twentieth century (AD 1896, 1933, 1968, 1994), and the 2011 Tohoku-oki tsunami^2^. Coinciding to these events, the coastal population and industry in these vulnerable regions has increased significantly over the past century^1^. This in context of an increased trust in protective measures (e.g., seawalls, tetrapod breakwaters)^3,4^ designed for earthquakes of smaller magnitude and misconceptions of existing tsunami models^5^ caused a general underestimation of the actual tsunami hazard^6,7^. Ultimately, the combination of hazard underestimation with the unexpectedly powerful 2011 Tohoku-oki earthquake (M_W_ 9.0^8^) and corresponding tsunami lead to the massive destruction and loss of life^4,7^.

Tsunami models prior to the 2011 event, which were based on the distribution of recognizable sand deposits, may have underestimated the hazard potential by the limitations of previous paleo- and historic tsunami studies to detect landward tsunami mud deposits^5^. The closest considered relative to the 2011 Tohoku-oki earthquake, the AD 869 Jogan earthquake and tsunami, was calculated with a magnitude of c. M_W_ 8.4^9–11^. These studies were all based on the detection and analysis of visually recognizable sand fraction of respective tsunami deposits. The sand unit of neither the AD 869 Jogan, nor the 2011 Tohoku-oki tsunami do spatially represent the full inundation^5,12^, leading to a misconception of the hazard potential. Studies following the 2011 tsunami suggest that previous estimates based on sedimentary evidence of paleo-tsunamis likely lead to the hazard underestimation^5,13^. Chagué-Goff et al.^12^ demonstrated that sandy deposits represent roughly 60% of the overall inundation for 2011 tsunami deposits at the Sendai Plain (Miyagi prefecture). While Abe et al.^14^ argued that the maximum extent of sandy deposits is linked to the total inundation distance. For the 2011 Tohoku-oki tsunami they report that for inundation distances of < 2.5 km sandy tsunami deposits can be observed up to 90% of this distance, while sediment deposits were observable only 50–76% when the total inundation distance was > 2.5 km^14^. Transferring this knowledge to the sedimentary record of past tsunami inundations along the same coast and considering other warning signals that have fallen into oblivion, such as tsunami stones and historical documents^3,15^, the underestimation of historic and paleo-tsunamis becomes evident.

It is true that small-scaled tsunamis can be too weak to cause considerable erosion, reworking, transport and redeposition of sediments and thus are unlikely to leave visually recognizable imprints in the sedimentary record^12,16^. This, however, may not to be confused with stratigraphically unrecognizable deposits beyond the sand limit during inundation or along locations with limited sediment sources and thus similarly appearing deposits (i.e., ‘invisible’ tsunami deposits). As Szczuciński et al.^17^ have documented for the Sendai Plain, only limited amounts of marine sediments have been transported on land during the 2011 Tohoku-oki tsunami and coastal sediments appear only up to 2.5 km inland, therefore ‘typical tsunami proxies’ may not be or only be applicable with limitations. The deposits beyond this point are mainly composed of eroded soils^18^ and following 2.9 km inland up to the inundation maximum (> 5 km) deposits contain no sand anymore^17^. In contrast to sandy tsunami layers, the deposits composed of eroded local soil will appear ‘invisible’ following a respective tsunami, and thereby contribute to a potential miscalculation of a tsunami’s magnitude. So far limited research into the ‘invisible’ sediment residues of tsunamis has been done. However, we can define three different types of ‘invisible’ tsunami deposits based on prior observations: (i) sandy tsunami deposits that have been (partly) eroded and thus become weak or entirely vanish; (ii) deposits beyond the limit to where the inundating waves are capable to carry sand or mud and only sea water inundated; and (iii) tsunami deposits that appear similar to the surrounding sediments based on their source material (e.g., soil erosion or sandy deposits at beaches). Judd et al.^16^ studied small-scaled tsunamis in Lyttelton Harbor (New Zealand) leaving behind atypical sediments without sand or even no sedimentary imprint at all. However, they were able, based on a multi-proxy approach, to attribute the discontinuous layers to the 1960 Chile and 1964 Alaska tsunamis. By the application of non-destructive techniques and radionuclide analysis, Shinozaki et al.^19^ detected in lake sediments from Hokkaido three invisible tsunami deposits using CT imaging, attributing to the 952 Tokachi-oki, 1960 Chile and 1973 Nemuro-oki tsunamis.

As analogous for historic and paleo-tsunamis, the study of modern tsunamis with known extents of the ‘invisible’ part of inundation from post-tsunami survey is needed. With this study we provide insight into yet undocumented woody-organic tsunami deposits of the 2011 Tohoku-oki tsunami from Misawa (northern Japan), in order to fill the knowledge gap and provide an understanding of hydrodynamic processes and preservation potential of these hardly recognizable tsunami residues.

## Setting

**Figure 2:**
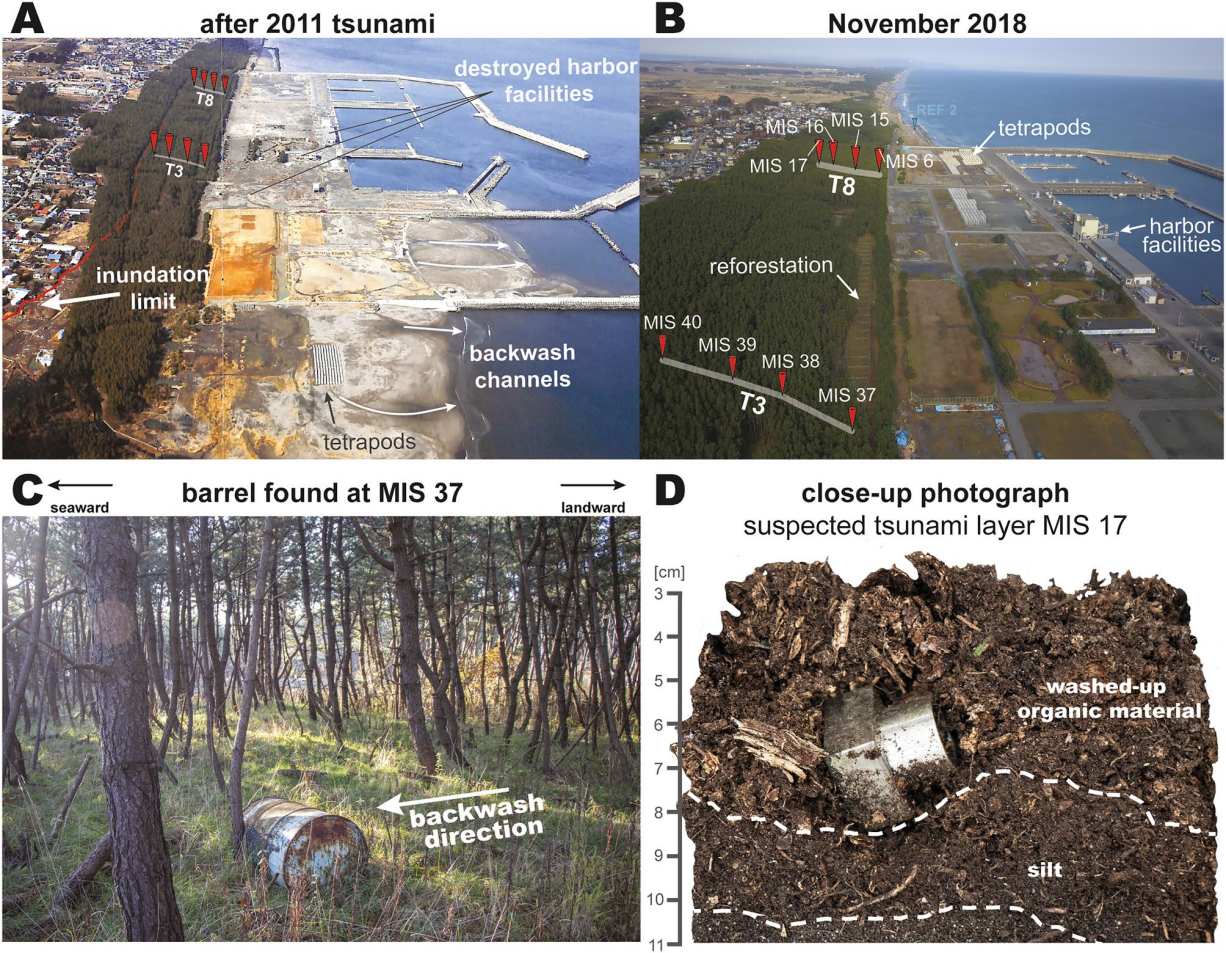
(**A**) Aerial photograph of the Misawa harbor directly after the 2011 tsunami showing the destruction and sediment distribution^24^. (**B**) Aerial photograph of the Misawa harbor during fieldwork in 2018 with the harbor rebuilt and reforestation efforts. (**C**) Barrel found in the forest at transect T3 leaning against a tree in backwash direction. (**D**) Close-up photograph of a suspected tsunami layer in MIS 17, consisting of washed-up organic material and containing a floatable plastic particle. Photographs were taken by author (Piero Bellanova) and photograph plate was illustrated using Adobe Illustrator (Creative Cloud version 2020, https://adobe.com/products/illustrator).

The Misawa harbor is located c. 320 km from the 2011 Tohoku-oki epicenter (Fig. 1A). Its coastal area is marked by an 100–200 m wide coastal lowland with c. 100 m long breakwaters (groins) every kilometer^20^. Coastal dune ridges are locally reinforced with concrete (since 2011) and a control forest (c. 150–250 m wide) consisting of black pines (*Pinus thunbergii*) covers the lowland constraining 10–20 m high terrace scarp (Fig. 1C). Parts of the control forest date back to the AD 1896 Meiji-Sanriku and 1933 Showa-Sanriku tsunami as a predecessing forest was heavily damaged and replanted^20,21^. Since 2011, the tsunami-damaged sections are subject to reforestation that causes the destruction of the 2011 tsunami’s stratigraphic legacy (Fig. 2).

The fishing port covers an area of c. 0.35 km^2^ and encompasses several harbor infrastructure facilities (fishing, oil tanks, etc.), three main piers and artificial sand piles for tetrapod production (Figs. 1B, 2). The recreational Miss Veedol beach, protected by seawalls, is located south of the main port area. Outside the southern greater port area, the small Mikawame creek flows into the sea incising the forest and dunes.

**Figure 3:**
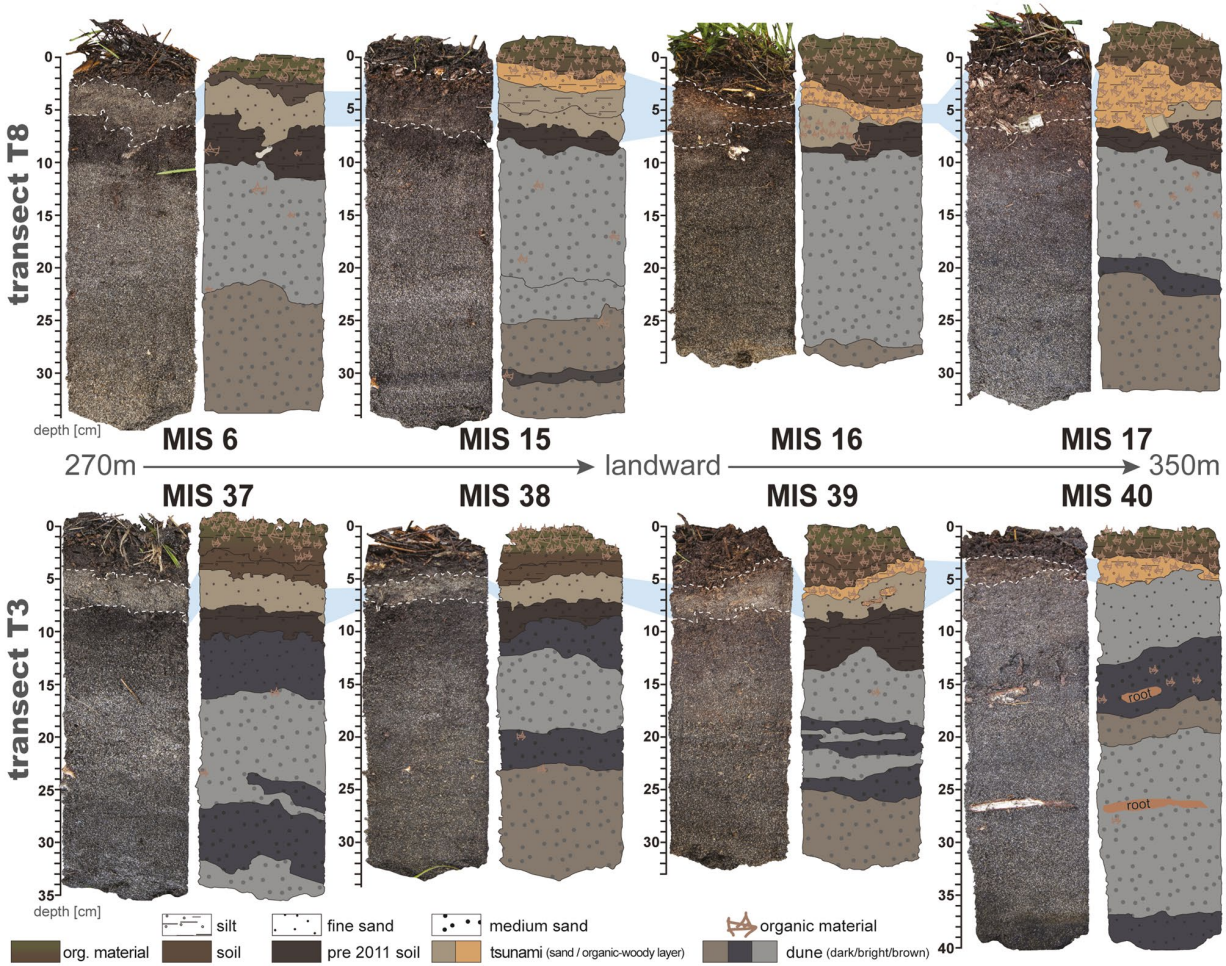
Photographs and visual representation of all stratigraphic profiles from transect T8 (top) and T3 (bottom). Profiles show thinning inland (left to right) of the 2011 tsunami sand deposits as well as a change in composition (to organic material). The tsunami deposits are marked by a blue band in the background, while upper and lower contacts of the tsunami deposits are indicated by dashed white lines. Shown data has been plotted using Microsoft Excel 365 (https://office.microsoft.com/excel) and is illustrated using Adobe Illustrator (Creative Cloud version 2020, https://adobe.com/products/illustrator).

The 2011 Tohoku-oki tsunami caused massive damages at the port of Misawa, for instance several fisheries docks have been teared off and drifted away by the water masses. Some of these floating docks have been found in the following years on the other site of the Pacific at the coasts of Oregon, Washington and Hawaii^22^. Almost the entire harbor area was covered with sand^23^ and debris, as well as onshore transported fishing trawlers^24^. Alongside, seawalls and oil tanks were damaged, and harbor structures flooded^24^. The tsunami run-up was locally measured with heights of 5.8 m at the port of Misawa^20^, while the maximum run-up height along the Misawa coast is estimated at 10.9 m^25^. At the studied field location at the Misawa harbor three tsunami waves were documented to inundate the coastal area^20,25^ with the furthest inundation in lower topography along the Mikawame creek reaching the residential areas (550 m from shore) and along the Misawa river (1700 m from shore)^25^, while the general inundation into the coastal forest ranged between 300 and 500 m. Within this coastal forest a total of eight intact soil strata samples from two coast-perpendicular transects were obtained by a Geoslicer device. Transect T3 spans over four locations from at c. 275 m to 350 m (sediment profile MIS 37–40, in the following are sample location names abbreviated as MIS for Misawa; Fig. 1C) inland and transect T8 from 275 to 350 m (MIS 6–17) (Figs. 1C, 2, 3; Table S1).

## Field observations and analytical results

### Field observations

Across the coastal control forest along the Misawa harbor several remnants from the 2011 Tohoku-oki tsunami inundation, such as barrels, fishing nets and buoys, have been found during field work in 2018. Nets partly indicated flow heights as they were stuck in tree crowns, while found barrels and buoys, partly buried by sediment and plant material, were situated at the landward-facing side (leeward) of tree trunks indicating flow directions (at site MIS 37; Fig. 2c). Several hundred meters inland reaching continuous sandy sediments have been found within the coastal control forest adjacent to the Misawa harbor. Debris within the coastal control forest was mainly found at surveyed locations in the vicinity of the harbor (50–100 m inside the forest), south of the Mikawame creek less debris was found until the Momoishi-Industrial Park (Oirase; c. 6 km south of the harbor). These observations accord to the described debris within the forest north of the harbor^20^. However, within the parts of the forest subject to reforestation, all traces (objects and sediments) of the 2011 tsunami have been cleared.

The stratigraphy of the Geoslicer profiles within the forest contains the former coastal dune sands overlaid by a humic soil developed by the forest. The dune sediments consist mainly of sand with varying coloration and corresponding content of silt or heavy minerals, as well as varying degrees of compactions and bioturbation (roots). Within or above the dark brown and organic-rich soil, a continuous sand layer laid by the 2011 Tohoku-oki tsunami contrasts trough sharp bottom contacts from the stratigraphy (Fig. 3). The stratigraphy reflects observations by Nakamura et al.^20^ north of the harbor area.

In transect T3 this tsunami sand layer starts in the most seaward Geoslicer profile with a thickness of c. 3 cm and shows a subsequent inland thinning until its disappearance between 340 and 350 m inland. Coinciding with the sand layer’s disappearance a bright brown layer of woody and organic material appears starting from c. 300–330 m inland.

In the northern transect T8 comparable observations in the stratigraphy have been made. Here, the tsunami sand layer is disappearing at c. 350 m inland and is only discontinuously present in MIS 17. Likewise, the tsunami sands tend to thin inland starting from c. 3 cm at MIS 6. Also, the woody and organic layer observed in transect T3 is found in this and additional transects surveyed along the Misawa coast. This organic layer occurred first in MIS 15 (c. 300 m from the shoreline) and increases in thickness to MIS 17, however, it disappears at c. 400 m inland. At the contact between the sand layer and the woody/organic layer floatable plastic particles have been found embedded in the sediment (Fig. 2D). In both transects fresh plant material (conifer needles, tree branches, grass) and soil covers the forest floor and thus form the top layer within each sample location’s stratigraphy.

### Granulometric data

**Figure 4:**
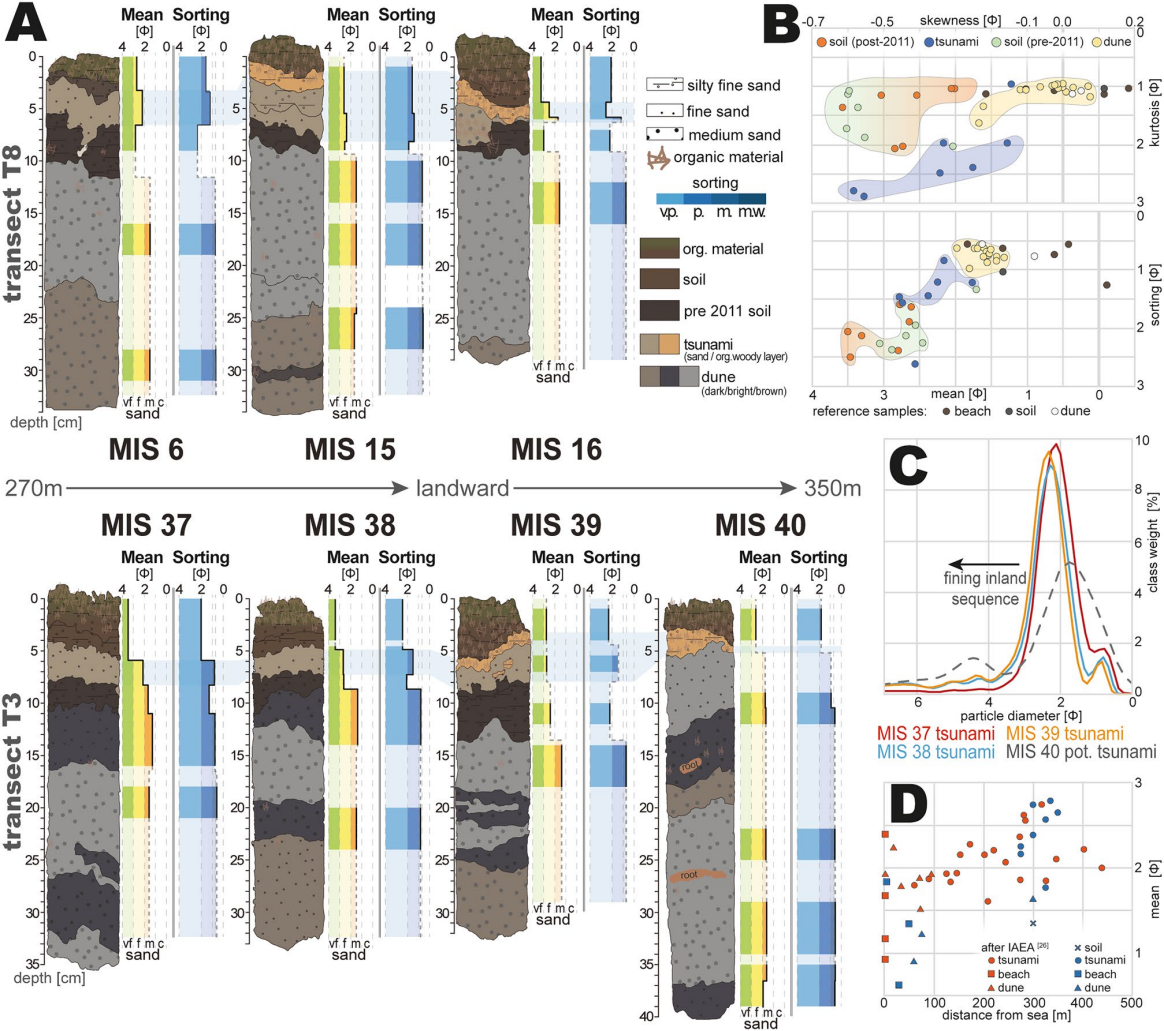
(**A**) Grain size distribution and sorting (Φ) in transects T3 and T8; (**B**) Bivariate plots of skewness against kurtosis and mean grain size against sorting (all in Φ) for all samples. Areas show grouping of soil, dune and tsunami samples; (**C**) Representation of the tsunami layer’s fining inland sequence in transect T3; (**D**) relationship between the distance from the shoreline and mean grain size of tsunami deposits and reference samples^26^. Orange symbols represent data from 4 transects at Misawa^26^ and blue symbols represent data from transect T3 and T8. Shown data has been plotted using Microsoft Excel 365 (https://office.microsoft.com/excel) and illustrated Adobe Illustrator (Creative Cloud version 2020, https://adobe.com/products/illustrator).

The dune sediments in both transects (T3 & T8, Fig. 4A) are comprised of moderately to moderately-well sorted medium sands (mean grain size 1.32–1.98 Φ). The soil layers under- and overlying the 2011 tsunami deposit are comprised of poorly to very poorly sorted, medium to (very) fine sands (1.71–3.47 Φ) with high amounts of mud (9–37%) (see Table S2 in the supplementary material). Contrasting from the soil, the 2011 tsunami sand deposit consists of mainly medium to fine sand with a coarser mean grain size (Fig. 4B). The skewness vs. kurtosis shows a separation between the strata-groups with the dunes being the most symmetrical and almost all mesokurtic, the soils being the finest skewed while being mesokurtic to leptokurtic, and the tsunami layer reflecting a mixture of both with fine skewing and very leptokurtic properties. Two outliers can be found with a woody tsunami sample (MIS 16) appearing similar to the dunes and the soil, as well as a pre-tsunami soil sample (MIS 37) within the field of tsunami samples (Fig. 4B). The latter is likely an effect of bioturbation by the roots mixing both the dunes and soil layers. Analogous differentiation between the three groups can be made by the mean vs. sorting (Fig. 4B). Dunes show the best sorting and the coarsest mean grain sizes, while the soils are represented by the smallest grain sizes and the poorest sorting. Tsunami sediments represent a mixture of different local sediment sources, such as the soil, dune and nearshore marine sediments (beach sand), and thus can be found in between both groups (Fig. 4B). Beach sands contribute to a high amount to the tsunami deposits within the first 200 m of the harbor area^26^ (Fig. 4D). These deposits feature high abundancies of heavy minerals, such as magnetite, that are decreasing inland^20,25,26^. The outliers show also compositional shifts, with the MIS 37 soil being near the dune sediments as a likely result of bioturbation, and the woody tsunami deposit of MIS 16 reflecting a mixture of local soil and dune sediments as its source material. An inland fining can be identified in transect T3 (Fig. 4C) for the 2011 tsunami deposit until the disappearance of sand between MIS 39 and MIS 40 (c. 340–350 m from the sea).

### Organic geochemical markers

**Figure 5:**
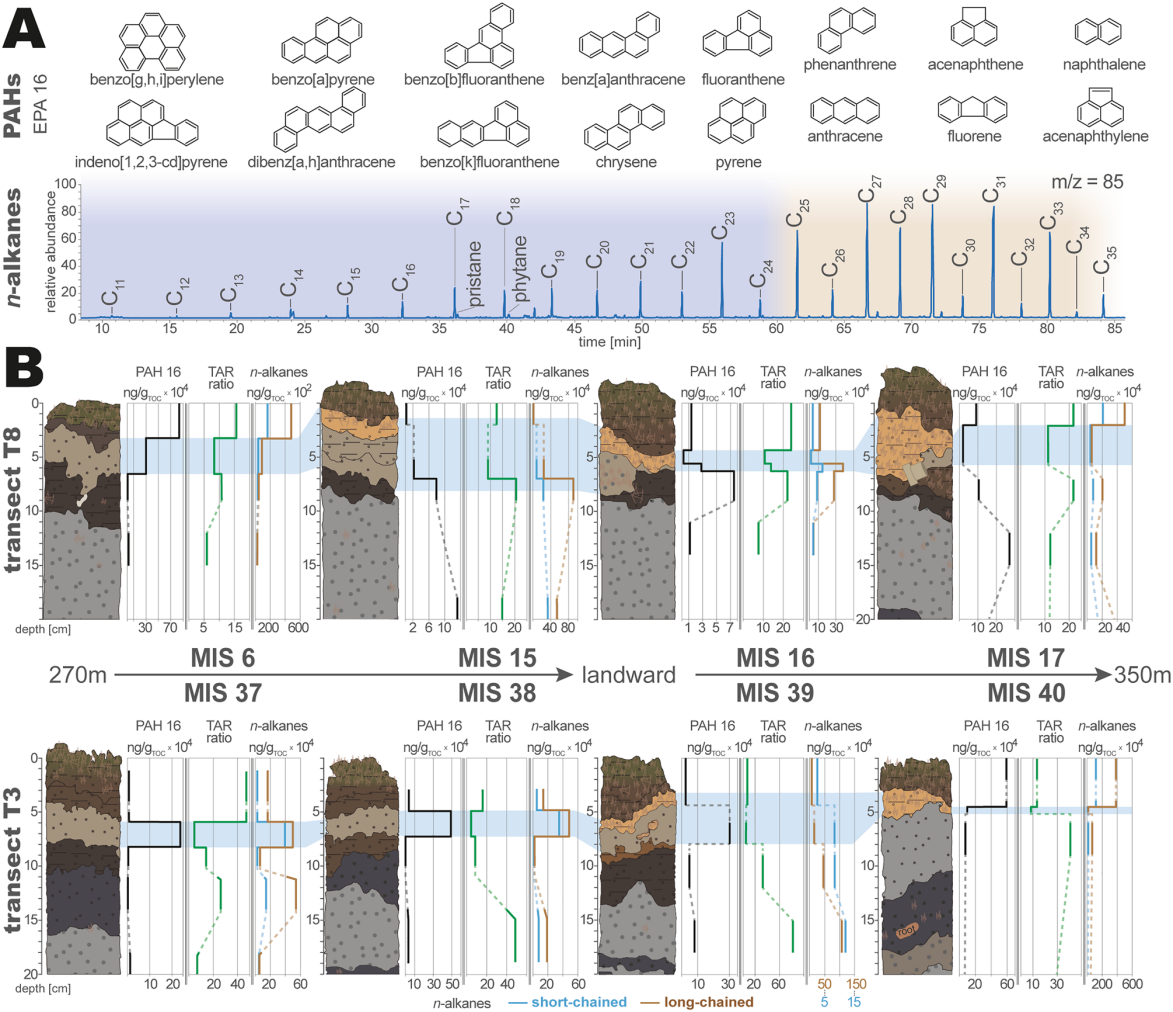
(**A**) Identified and quantified organic proxies (PAHs and *n*-alkanes); (**B**) Concentration profiles of PAHs (black), terrigenous/aquatic ratio (TAR—green) and the total short-chained and terrestrial long-chained *n*-alkane concentrations (blue and brown) within each sedimentary profile of both transects. Shown data has been plotted using Microsoft Excel 365 (https://office.microsoft.com/excel) and illustrated Adobe Illustrator (Creative Cloud version 2020, https://adobe.com/products/illustrator).

A variety of organic geochemical proxies, both biological and anthropogenic, have been consulted for the determination of sediment sources and pollution through the harbor destruction during the 2011 Tohoku-oki tsunami. As biological markers *n*-alkane distributions and corresponding ratios have been quantified (Fig. 5A). Each sample’s composition of different chain-length *n*-alkanes provides insight into sediment sources. Shorter-chained *n*-alkanes (C_25_ <) hereby represent the ubiquitous biomass production (both marine and terrestrial), while longer chain lengths (> C_25_) are produced exclusively by terrestrial higher land plants (e.g., cuticular waxes)^27,28^. All samples across the studied transects reflect a high bioproductivity in the coastal environment by the high abundance of ubiquitous short-chained *n*-alkanes. Higher concentrations in the C_27_ and C_29_ *n*-alkanes correlating to leave waxes of the black pines (*Pinus thunbergii*^29^) can be observed in all samples. In MIS 6 the highest amounts of short- and long-chained *n*-alkanes are in the topsoil, while in MIS 15 the highest amounts of short- and long-chained *n*-alkanes are in the dune samples. Here the 2011 tsunami layer is likely comprised of a mixture of sediment sources from the nearshore, beach and the eroded forest floor itself. Further inland at MIS 16, the tsunami sand layer stands out by representing the highest concentration of short-chained and long-chained terrestrial *n*-alkanes within this stratigraphic profile. On the contrary, the woody and organic layer of MIS 16 separates itself by the lowest amounts of both short- and long-chained *n*-alkanes. In MIS 17 with the floating plastic particle the tsunami layer represents slightly lower concentrations from the over- and underlying soil.

The sediment profiles located closest to the coast of transects T3 and T8 contrast each other significantly in the contribution of short-chained *n*-alkanes within their 2011 tsunami layer. In profiles MIS 37 to MIS 38 the tsunami layer has the significantly highest short-chained *n*-alkane concentration, only in MIS 40 where the sand deposit disappeared no strong indication of short-chained *n*-alkanes is visible. The terrigenous/aquatic ratio (TAR), an indicator to determine relative amounts of terrestrial versus aquatic *n*-alkanes in a sediment^30^, responds in relation to the total long-chained and short-chained *n*-alkane concentration. In most tsunami samples across both transects, the TAR can be used to differentiate the tsunami layer from the surrounding terrestrial sediments through a lower terrestrial and increased marine signal.

In order to grasp the pollution caused by the Misawa harbor’s destruction during the 2011 Tohoku-oki tsunami the 16 EPA-priority pollutant polycyclic aromatic hydrocarbons (PAHs) have been evaluated. Their concentrations within the forest’s stratigraphy reflect a clear indication of pollutant input in the past, however, the results for the 2011 tsunami sand deposit vary significantly between the two studied transects (Fig. 5B). In transect T3 (with the exception of sediment profile MIS 40) the concentration of PAHs in the tsunami sand layer significantly exceeds that of the surrounding deposits. The destruction in the harbor area, especially the washed away heavy oil tanks, ships and barrels^24^, can be associated with the pollution in the transect T3 tsunami layer. In transect T8, however, the pre-2011 soil (MIS 16) or dune sediments (MIS 15 & 17) represents the highest concentrations. This is either ascribable to a seeping effect from the tsunami sand into the finer soil, as described by Shinozaki et al.^31^ for marine biomarkers at the Sendai Plain; or (especially for the dune deposits) a pre-existing pollution accumulating over a longer period of time.

## The unrecognized tsunami deposits

### 2011 Tsunami deposits in Misawa

Frequent observations recognized following major tsunamis, such as scours on the leeward sides of trees, inland transported objects (ships, buoys, fishing nets, barrels, etc.) and a several hundred meters inland reaching wash over fan of sandy sediment, have been found within the coastal control forest inland of the Misawa harbor. These sediment remnants of the 2011 Tohoku-oki tsunami have been observed across the Misawa harbor area after the tsunami (Fig. 2A;^23^) and now have been traced until their maximum transport distance at about 350 m inland within the coastal control forest (in 2018). The sandy tsunami deposit exhibits similar characteristics (e.g., thickness, inland fining and thinning, grain size, etc.) as sand layers described further north of the harbor^20,21,25^. The sediment sources can be mainly attributed to marine and coastal sediments^20^, as well as terrestrial material from the forest floor, based on the distribution of *n*-alkanes and the TAR ratio. However, the two studied transects are represented by varying sediment sources, as transect T3 located closer to the Miss Veedol beach and thus is dominated by unconsolidated coastal and marine sediment sources, a stronger marine influence is detectable within the transects sandy tsunami deposits. On the contrary is transect T8 located towards the northern end of the harbor facing the fortified harbor area and relatively steeper topography (Fig. 1C) with less mobile marine sediments available for erosion. This is reflected in transect T8′s tsunami layer with a more dominant terrestrial signature, which is nonetheless still contrasting from the forest soil, especially in the TAR ratio. In a similar fashion is the destruction caused by the 2011 tsunami echoed by the PAH pollution in the transects. The PAH concentration in transect T3 is significantly higher and more distinct within all sandy tsunami layers, compared to transect T8. The pollution is consequently location-specific, as most of the harbor’s facilities are located in front of transect T3 (Fig. 2A; Fig. S1B,D) and coinciding most of the large debris (e.g., lacquer barrels) has been found near MIS 37. The harbor area near transect T8 was not much developed and served as a storing ground for concrete tetrapods. Thus, the lack of pollution sources is likely the reason for the lower overall PAH pollution in the tsunami layer. The soils beneath T8′s tsunami deposits present in parts higher concentrations of PAHs, which are either a result of long-term accumulation of atmospheric PAH’s or the result of short-term seeping of pollutants from the coarse sand into the finer soil. However, based on the immobility of these compounds the latter is rather unlikely. In combination with other datasets from the Aomori coast^20,21,25,26^ the effect of the 2011 Tohoku-oki tsunami for the northern Japanese coastline can be depicted.

While the detection of the sandy tsunami deposits across the forest is straight forward based upon reports, field observations and analytical results, the detection of the woody and organic tsunami layer composed of eroded forest material and transported further inland than the sand layer is more complex. Even though Nakamura et al.^20^ described that the 2011 tsunami deposit was covered by leaves that have been stripped from trees by the seawater inundation, no further analyses or descriptions of this layer have been made. At first, this layer was not apparent, neither during field work, nor in the later acquired analytical data. The discovery of floatable plastic particles in MIS 17 embedded within this woody and organic material (Figs. 2D, 3), however, changed the interpretation as the brighter and finer organic layer must have obviously be the result of the inundating water masses inside the forest.

### Inundation and backwash effects

**Figure 6:**
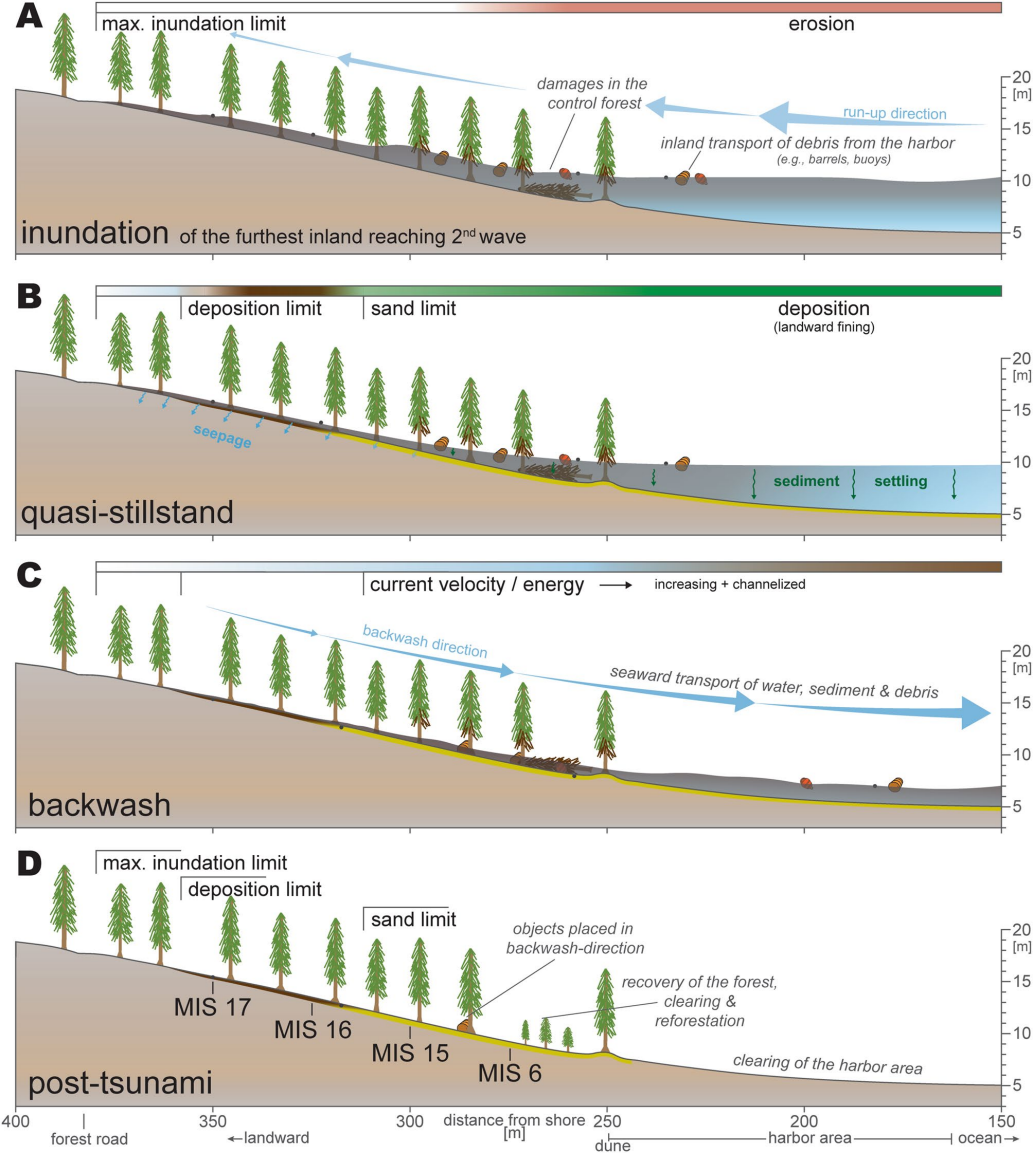
Conceptual model of erosion, transport and deposition of the sandy and organic-woody tsunami deposits during the 2011 Tohoku-oki tsunami at the Misawa harbor. (**A**) Inundation and erosion within the forest; (**B**) quasi-stillstand with settling and deposition of eroded material; (**C**) downslope increasing backwash with increasing erosion-potential and channelization; (**D**) post-tsunami setting and coring sites exemplary on transect T8. The conceptual model has been illustrated Adobe Illustrator (Creative Cloud version 2020, https://adobe.com/products/illustrator).

A total of three waves inundated the Misawa harbor according to eyewitness accounts, sequential photographs and supported by tide gauge data from nearby Hachinohe^21,25^. The first wave reached the Misawa harbor approx. 35 min after the earthquake. The second wave reached the maximum inundation height approx. 2 h following the earthquake. As the second wave had the highest estimated flow speed, inundation height and inundated furthest inland^25^, it is considered to have had the most destructive power within the harbor area and deposited most of the sedimentary remnants. The third wave has been documented to be the smallest of the set and likely did not leave sedimentary traces behind^21,25^. Sediments laid by the first wave have been at least in part subject to erosion by the second wave. Approximately 1.75 km south of the harbor area thicker and sub-layered deposits can be found indicating the reported three tsunami waves^20,25^. Nonetheless, an unconventional differentiation between sandy tsunami deposit and organic-woody material eroded from the forest floor, transported inland and deposited on top of the sand and even beyond the limit of sand has been recognized. This layer is interpreted to be the result of the second wave reaching the maximum inundation distance within the forest which is supported by the embedded plastic piece (Fig. 6A). As the woody and organic material is lighter and subject to floating, it is transported further inland as lower energy is required for its transport and settling on top of the sand when current velocities drop (quasi-stillstand; Fig. 6B). Likewise, its thickness is at first increasing with increasing erosion of the forest floor.

Along the Aomori coast and Misawa varying reports about the occurrence of a backwash have been documented for the 2011 Tohoku-oki tsunami. While Nakamura et al.^20^ could not document any evidence for a backwash or only a very gentle non-erosive or non-sediment-transporting backwash, Koiwa et al.^25^ document the backwash phenomenon at the Misawa river with photographs and field observations (e.g., bent grass, backwash fan-like lobes). Our field observations of leeward-oriented (backwash-direction) on trees leaning barrels (Fig. 2C) support a backwash current for the Misawa harbor with the topographical setting being the main driver for the water to retreat (Figs. 1C, 6C). Further do aerial photographs of the Misawa harbor suggest at least four small channels towards the ocean caused by the channelized seaward flowing water masses (Fig. 2A). This, however, occurs only in the non-consolidated and less fortified southern part of the harbor near the Miss Veedol beach and the Mikawame creek. The beaches have recovered since the 2011 Tohoku-oki tsunami, not featuring any backwash channels anymore (Fig. S1A & B), thus also excluding their origin from tidal variation (c. 1 m tidal range at Misawa harbor). The sedimentary imprint, especially the woody-organic layer is only partially affected by the backwash. Near the maximum inundation the volume of inundated water is lower, as is the inundation height. Here the water level further decreased during quasi-stillstand through seeps into the coarse ground (Fig. 6B). After the quasi-stillstand the water retreated only slowly as the backwash currents energy is initially low but slowly increasing based on the topography, thus not causing a strong enough current to erode sediments deposited furthest inland (Fig. 6C). This suggests that the return flow in the furthest inland parts of the inundated areas was gentle in comparison with run-up and did not transport much sediment, similar to the observations by Nakamura et al.^20^. However, downslope the current velocity increases and with it the backwash’s capability to erode previously by the inundation deposited sediments (Fig. 6D). This is apparent, as the woody and organic tsunami layer is missing in seaward parts of the forest (300–280 m inland). The variation in tsunami sand and woody-organic layer between both transects, such as the observed distances and thicknesses, may be linked to the location-specific backwash direction, topographical features (e.g., a small dune crest in front of the forest), and a trend to channelization, leading to different erosive behavior. Also, the preserved sand thickness deposited by the tsunami at the Misawa harbor is considerably smaller compared to documented thicknesses exceeding 10 cm in locations with a flat topography along the Aomori coast^20,21,25^. This variation in observed tsunami sand thickness may be correlated to the preservation, as transects were surveyed seven years following the tsunami. Further, does this observation corresponds with the found barrels, concluding in a downhill increasing erosive backwash that ultimately flows channelized back into the ocean (Fig. 6C). Furthermore, have most transects within the harbor area only been affected by the strongest second inundation and resulting backwash, therefore many features of only this wave have been preserved in the sedimentary record.

## Preservation potential and implications

### Preservation potential

Sandy tsunami deposits are favored by (paleo-)tsunami research over muddy deposits based on their preservation and detectability in the sedimentary record. The observation of the woody-organic tsunami layer at the Misawa harbor, will likely not change this. As this layer has been and will be subject to natural organic alterations, such as pedogenesis^32^; in a few years a clear differentiation between the post-tsunami accumulated soil and the by the tsunami eroded, transported and deposited organic forest material will be difficult. It is also highly likely that this process combined with bioturbation already altered the layer since the 2011 tsunami^26^ until field work took place in 2018. Therefore, long-term monitoring of these deposits is needed to gain a better understanding of the preservation behavior of mostly organic material deposits laid by modern tsunamis to learn for the assessment of historic and paleo-tsunamis. Here potentially organic geochemical markers may be favored in the future with the capability to preserve the immobile geochemical imprint over longer periods^18^.

Especially along the Aomori coast, but a phenomenon occurring globally is the anthropogenic alteration of deposits. In Misawa and observed along the entire Aomori coast the reforestation effort (Fig. 1B) and the construction of new seawalls and other protective measures are likely needed for future tsunami mitigation. This, however, destroys the sedimentary archives irrevocably.

### Implications of the woody-organic tsunami layer

The identification of yet unrecognized processes and sedimentary remnants of tsunamis is of importance for tsunami research. The 2011 Tohoku-oki tsunami is the best documented and studied modern tsunami, and the intensive study of the past decade led to several new insights improving our understanding of tsunami processes and transferring this knowledge to paleo-tsunami research. The best example for this improved understanding may be the discovery following the inundation of the Sendai plain (Japan). Here Abe et al.^14^ and Chagué-Goff et al.^12^ documented that the tsunami sand deposits covered only about 50–76% but muddy deposits up to 95% of the inundated distance (c. 4.85 km). Even if this percentage is unlikely to be applicable to every tsunami as these events are highly depending on topographical and other features, it leads to the conclusion that past studies unintentionally may have underestimated a tsunamis magnitude by only studying the sandy deposits.

For areas north of the Misawa harbor Nakamura et al.^20^ document tsunami sands reaching near the inundation limit, which can be attributed to the flatter topography. At the Misawa harbor the tsunami sands reach c. 340–350 m inland representing c. 61–63% of the reported total inundation. This corresponds to observations^12,14^ from the Sendai Plain. However, the detected woody-organic tsunami deposit exceeds the tsunami sands by c. 40–60 m inland or 69–72% of the total inundation.

Initially the organic and woody layer was not evident during early stages of the field work and this would have been unchanged without the discovery of the floatable plastic particles in MIS 17. That critical observation was the turning point for the interpretation of the layer’s origin and thus our understanding of processes during the Tohoku-oki tsunami in the coastal control forest at the Misawa harbor. Geochemical approaches (e.g., organic geochemistry) may lead the way into further insights of these hardly recognizable or invisible tsunami deposits in the future. Hereby, can geochemical data and knowledge about modern hardly recognizable tsunami deposit examples (e.g., wood-organic deposits from Misawa) be transferred, in consideration of the preservation potential, to paleo- and historic tsunami deposits, where these fragile remnants are most certainly vanished from the naked eye.

Furthermore, are observations of backwash processes consecutively improving our knowledge about this phenomenon, as it is still relatively underrepresented in tsunami studies. Overall, may the observation of the woody-organic and up to now undocumented layer lead to an improvement in the understanding of tsunami processes. The detection of the woody-organic tsunami deposits presented in this study can in view of observations of sand-limits^12,14^ and atypical deposits^16^, also be transferred to tsunamis inundating less anthropogenic influenced natural environments, such as low-lying coastal wetlands. This knowledge from modern tsunami deposits may be transferable to paleo-tsunami research, and thus to a revised coastal hazard assessment not only for the Japanese coastline.

## Methods

### Sampling

During fieldwork, a total of 59 intact sedimentary Geoslicer profiles from 13 coastal perpendicular transects spanning over a coastal stretch of c. 8 km (Misawa to Oirase) were obtained and labeled MIS (for the location Misawa coast) and the subsequent sample location number (Table S1). For the transects T3 and T8 a duplicate set with a total of 41 sediment samples was collected from eight Geoslicer profiles for grain size and organic geochemical analysis. For the latter, samples are placed in pre-cleaned aluminum trays to avoid contamination and refrigerated to limit microbial alteration. Samples were analyzed at the Neotectonics and Natural Hazards Group and Institute for Geology and Geochemistry of Petroleum and Coal (both RWTH Aachen University, Germany).

### Grain size analysis

Grain size samples were dried at 40 °C in a drying chamber (approx. 48 h) and sieved to < 2 mm. Dried samples were smoothly disintegrated using a mortar to avoid sediment aggregates. Organic matter is removed with hydrogen peroxide (H_2_O_2_, 15%) and coagulation limited by the addition of sodium pyrophosphate (Na_4_P_2_O_7_, 46 g/l^33^). Grain size measurement was performed with a LS 13320 Laser Diffraction Particle Size Analyzer (Beckman Coulter Inc., CA, USA) at the laboratories of the Institute of Geography (Cologne University, Germany). Contrary to the complex Lorenz-Mie theory^34^, that improves measurements of particles < 1 µm, the Fraunhofer optical model approximates the diffraction at the particle surface and thus is most appropriate for larger grain sizes^35^. Using the optical Fraunhofer model (0.4–2000 μm) according to ISO 13320^36^ all samples were measured three times to decrease the error margin. Grain size parameters were calculated using GRADISTAT software version 8^37^ based on Folk and Ward^38^.

### Total organic carbon (TOC)

Total organic carbon (TOC) determination was carried out using a liquiTOC II (Elementar Analysesysteme GmbH, Germany) in the single analytical run mode. 100 mg dried homogenized sample material was weighed into a quartz boat and measured.

### Extraction of organic geochemical compounds

Organic-geochemical compounds were extracted via solid–liquid extraction from the sediments using the overhead shaking extraction protocol after Bellanova et al.^18,39^. Wet sediment aliquots of 10–100 g were extracted with acetone and *n*-hexane as solvents. Extracts were separated, reduced in volume via rotary evaporation, dried over anhydrous sodium sulfate and desulphurized with activated copper.

All extracts were fractionated into six fractions via column chromatography (J.T. Baker, 2 g silica gel 40 mm) with eluents based on solvent mixtures of *n*-pentane, dichloromethane and methanol according to the protocol by Franke et al.^40^. Each sample fraction was spiked with 50 μl of internal surrogate standard solution (5.8 ng/μl fluoracetophenone, 6.28 ng/μl d_10_-benzophenone and 6.03 ng/μl d_34_-hexadecane).

### GC and GC–MS analysis

For qualitative identification and quantitative determination of concentration, standardized gas chromatography-mass spectrometry (GC–MS) was applied. GC–MS measurements were performed on a quadrupole Trace GC–MS (Thermo Finnigan LLC, USA) with helium as carrier gas, equipped with a 30 m × 0.25 mm i.d. × 0.25 μm film ZB-5 fused silica capillary column (Zebron capillary GC column, Chrompack). Samples were measured with a temperature program starting at 60 °C (injector temperature 270 °C) and a splitless time of 60 s, a heating rate of 3 °C/min to 310 °C with an isothermal time of 20 min. Measurements in full scan mode achieve a range from 35 to 650 m/z in positive electron impact ionization mode (EI +) with 7 eV electron energy.

Qualitative compound identification was achieved via the mass spectra NIST MS database (National Institute of Standards and Technology (NIST), U.S. Department of Commerce, USA); and the subsequent verification of mass spectral parameter was performed with standard reference materials. Quantitative quantification of compound concentrations was accomplished by integration of specific ion chromatograms in combination with external four-point calibration of reference material and a normalization to TOC.

## Supplementary Information

Supplementary Information.

## Data Availability

All data that have contributed to the reported results are available from the corresponding author at request.
