# Supplementary material for: Tracing woody-organic tsunami deposits of the 2011 Tohoku-oki event in Misawa (Japan)
Source: Sci Rep. 2021 Apr 26;11:8947. doi: 10.1038/s41598-021-88199-3 (PMC8076248; doi:10.1038/s41598-021-88199-3)
Supplement: Supplementary file 1 — Supplementary Information. [file 41598_2021_88199_MOESM1_ESM.docx]

**Supplementary material**

Tracing woody-organic tsunami deposits of the 2011 Tohoku-oki event in Misawa (Japan)

Piero Bellanova ^1,2*^, Mike Frenken ^1,2^, Yuichi, Nishimura ^3^, Jan Schwarzbauer ^2^, Klaus Reicherter ^1^

*^1^Institute for Neotectonics and Natural Hazards, RWTH Aachen, Lochnerstrasse 4-20, 52056 Aachen, Germany*

*^2^ Institute for Geology and Geochemistry of Petroleum and Coal, RWTH Aachen University, Lochnerstrasse 4-20, 52056, Aachen, Germany*

*^3^ Institute of Seismology and Volcanology, Hokkaido University,* *Kita-10, Nishi-8, Kita-ku, 060-0810 Sapporo, Japan*

*(*Corresponding author,* email: p.bellanova@nug.rwth-aachen.de*)*

*Submitted to Nature Scientific Reports*

# Figures

Figure S1: Plate of overview photograph from Misawa harbor. (A) MissVeedol beach recovered from backwash channels. (B) MissVeedol beach recovered from backwash channels and harbor recovered from tsunami damages. Transect T1, T2, reference samples 5 & 6, and studied transect T3 are located in the coastal control forest. (C) Mikawame creek south of the harbor featuring newly constructed tsunami walls and reforestation. Also the location of the reference samples are indicated. (D) Location of transect T3 with potential sources on the harbor area from were found debris (buoys, barrels, nets, etc.; Fig. 2C) could originate. Photographs were taken by author (Piero Bellanova) and photograph plate was illustrated using Adobe Illustrator (Creative Cloud version 2020, https://adobe.com/products/illustrator).


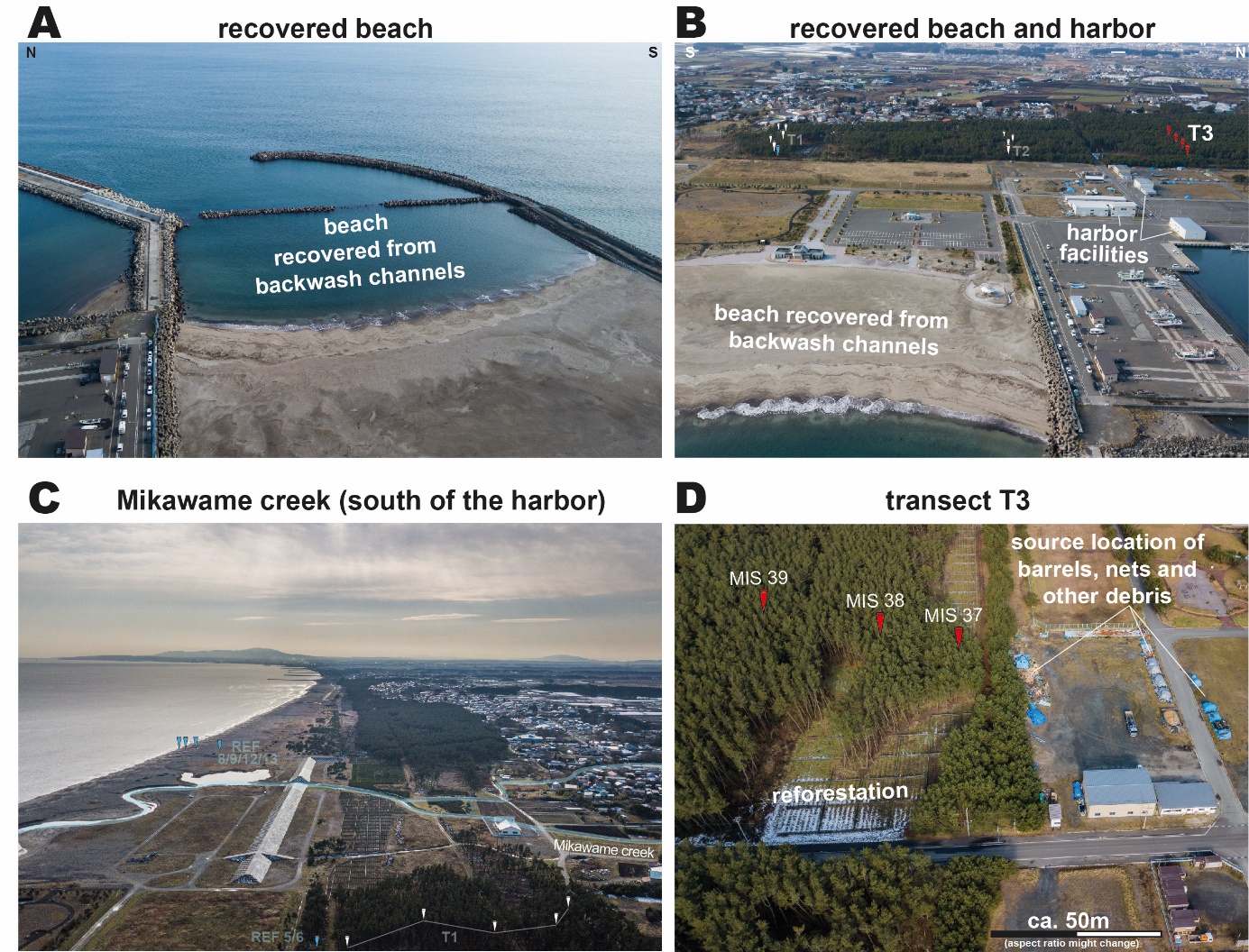
Figure S1

# Tables

Table S1: GPS location and elevation of the surveyed transects and reference samples.

Table S2: Granulometry results after Folk and Ward ^[38]^ in phi [Φ] and descriptive, as well as the percentual distribution of sand and mud for transect T3 (MIS 6, MIS 15 and MIS 16) and transect T8 (MIS 37, MIS 38, MIS 39, MIS 40).

Table S3: Organic geochemical results in ng/g_TOC_ for transect T3 (MIS 6, MIS 15, MIS 16 and MIS 17) and transect T8 (MIS 37, MIS 38, MIS 39, MIS 40).

Table S4: Granulometry results for reference samples after Folk and Ward ^[38]^ in phi [Φ] and descriptive, as well as the percentual distribution of sand and mud.

| **Geoslicer** | **coordinates** | **transect** | **elevation**  **[m.a.s.l.]** | **Distance to coast [m]** |
| --- | --- | --- | --- | --- |
| MIS 6 | 40°40'59.22"N 141°25'52.62"E | T8 | 10 | 275 |
| MIS 15 | 40°40'59.22"N 141°25'51.25"E | T8 | 12 | 300 |
| MIS 16 | 40°40'59.22"N 141°25'50.16"E | T8 | 13.5 | 325 |
| MIS 17 | 40°40'59.40"N 141°25'49.32"E | T8 | 15 | 350 |
| MIS 37 | 40°40'42.90"N 141°25'57.72"E | T3 | 8 | 275 |
| MIS 38 | 40°40'42.60"N 141°25'56.58"E | T3 | 10 | 300 |
| MIS 39 | 40°40'42.66"N 141°25'54.96"E | T3 | 12 | 335 |
| MIS 40 | 40°40'42.48"N 141°25'54.36"E | T3 | 13 | 350 |
| MIS REF 2 | 40°41'19.52"N 141°25'51.00"E | beach | 0 | 5 |
| MIS REF 5 | 40°40'28.38"N 141°26'3.72"E | soil | 7 | 300 |
| MIS REF 6 | 40°40'28.38"N 141°26'3.72"E | palaeodune | 7 | 300 |
| MIS REF 8 | 40°40'12.60"N 141°26'17.76"E | beach | 2 | 30 |
| MIS REF 9 | 40°40'12.72"N 141°26'18.54"E | beach | 1 | 10 |
| MIS REF 12 | 40°40'12.72"N 141°26'19.14"E | beach | 0 | 0 |
| MIS REF 13 | 40°40'12.36"N 141°26'16.50"E | dune | 4 | 60 |
| MIS REF 24 | 40°39'31.20"N 141°26'28.50"E | beach | 3 | 50 |

Table S1

|  | Mis 6 | | | | | Mis 15 | | | | | | Mis 16 | | | | |  |  |  |
| --- | --- | --- | --- | --- | --- | --- | --- | --- | --- | --- | --- | --- | --- | --- | --- | --- | --- | --- | --- |
| depths [cm] | 0-2 | 2-5 | 5-9 | 16-19 | 28-31 | 1-5 | 5-6 | 6-9 | 10-14 | 16-20 | 24-28 | 0-3 | 3-4 | 4-6 | 7-9 | 12-16 |  |  |  |
| description | top soil | tsunami | soil | dune | dune | top soil | tsunami | soil | dune | dune | dune | top soil | tsunami | tsunami | soil | dune |  |  |  |
| mean [Φ] | 2.77 | 2.25 | 2.89 | 1.54 | 1.57 | 2.62 | 2.38 | 2.56 | 1.53 | 1.51 | 1.69 | 3.31 | 2.56 | 1.77 | 3.06 | 1.61 |  |  |  |
| sortig [Φ] | 1.60 | 1.22 | 2.38 | 0.76 | 0.69 | 1.64 | 1.45 | 1.95 | 0.73 | 0.66 | 0.64 | 2.14 | 2.62 | 1.23 | 2.27 | 0.79 |  |  |  |
| skewness [Φ] | 0.47 | 0.25 | 0.60 | 0.02 | 0.00 | 0.45 | 0.34 | 0.55 | 0.10 | 0.03 | 0.00 | 0.31 | 0.14 | 0.33 | 0.60 | -0.08 |  |  |  |
| kurtosis [Φ] | 2.06 | 2.37 | 1.07 | 0.98 | 0.97 | 2.01 | 2.46 | 1.87 | 1.07 | 0.99 | 1.01 | 1.02 | 0.95 | 1.97 | 1.11 | 1.18 |  |  |  |
| mean | F. Sand | F. Sand | F. Sand | M. Sand | M. Sand | F. Sand | F. Sand | F. Sand | M. Sand | M. Sand | M. Sand | V.F. Sand | F. Sand | M. Sand | V.F. Sand | M. Sand |  |  |  |
| sortig | Poorly | Poorly | V. Poorly | Mod. | Mod. Well | Poorly | Poorly | Poorly | Mod. | Mod. Well | Mod. Well | V. Poorly | V. Poorly | Poorly | V. Poorly | Mod. |  |  |  |
| skewness | V. Fine | Fine | V. Fine | Sym. | Sym. | V. Fine | V. Fine | V. Fine | Fine | Sym. | Sym. | V. Fine | Fine | V. Fine | V. Fine | Sym. |  |  |  |
| kurtosis | V. Leptok. | V. Leptok. | Mesok. | Mesok. | Mesok. | V. Leptok. | V. Leptok. | V. Leptok. | Mesok. | Mesok. | Mesok. | Mesok. | Mesok. | V. Leptok. | Leptok. | Leptok. |  |  |  |
| sand [%] | 82 | 90 | 73 | 98 | 99 | 83 | 87 | 81 | 97 | 99 | 99 | 63 | 69 | 91 | 72 | 98 |  |  |  |
| mud [%] | 18 | 10 | 27 | 2 | 1 | 17 | 13 | 19 | 3 | 1 | 1 | 37 | 31 | 9 | 28 | 2 |  |  |  |
|  |  |  |  |  |  |  |  |  |  |  |  |  |  |  |  |  |  |  |  |
|  | Mis 37 | | | | | Mis 38 | | | | | Mis 39 | | | | Mis 40 | | | | |
| depths [cm] | 0-4.5 | 4.5-8 | 8-12 | 12-16 | 18-21 | 0-4 | 4.5-6.5 | 6.5-10 | 10-14 | 20-24 | 1-4 | 5.5-7 | 10-12 | 14-18 | 1-4 | 8-12 | 22-25 | 29-34 | 35-39 |
| description | top soil | tsunami | soil | dune | dune | top soil | tsunami | soil | dune | dune | top soil | tsunami | soil | dune | top soil | dune | dune | dune | dune |
| mean [Φ] | 3.50 | 2.16 | 1.71 | 1.32 | 1.60 | 3.47 | 2.74 | 2.70 | 1.43 | 1.55 | 2.80 | 2.78 | 2.46 | 1.43 | 2.65 | 1.81 | 1.74 | 1.66 | 1.98 |
| sortig [Φ] | 2.05 | 0.85 | 1.34 | 0.80 | 0.64 | 2.50 | 1.57 | 2.14 | 0.85 | 0.89 | 2.39 | 1.47 | 2.26 | 0.78 | 1.89 | 0.99 | 0.64 | 0.60 | 0.64 |
| skewness [Φ] | 0.61 | 0.16 | 0.31 | -0.03 | -0.07 | 0.41 | 0.55 | 0.60 | 0.22 | 0.12 | 0.30 | 0.58 | 0.57 | 0.02 | 0.51 | 0.23 | 0.01 | 0.06 | -0.01 |
| kurtosis [Φ] | 1.36 | 1.96 | 2.02 | 1.02 | 1.00 | 1.13 | 2.87 | 1.71 | 1.32 | 1.04 | 1.02 | 2.77 | 1.36 | 0.98 | 1.13 | 1.61 | 1.01 | 1.01 | 1.09 |
| mean | V.F. Sand | F. Sand | M. Sand | M. Sand | M. Sand | V.F. Sand | F. Sand | F. Sand | M. Sand | M. Sand | F. Sand | F. Sand | F. Sand | M. Sand | F. Sand | M. Sand | M. Sand | M. Sand | M. Sand |
| sortig | V. Poorly | Mod. | Poorly | Mod. | Mod. Well | V. Poorly | Poorly | V. Poorly | Mod. | Mod. | V. Poorly | Poorly | V. Poorly | Mod. | Poorly | Mod. | Mod. Well | Mod. Well | Mod. Well |
| skewness | V. Fine | Fine | V. Fine | Sym. | Sym. | V. Fine | V. Fine | V. Fine | Fine | Fine | V. Fine | V. Fine | V. Fine | Sym. | V. Fine | Fine | Sym. | Sym. | Sym. |
| kurtosis | Leptok. | V. Leptok. | V. Leptok. | Mesok. | Mesok. | Leptok. | V. Leptok. | V. Leptok. | Leptok. | Mesok. | Mesok. | V. Leptok. | Leptok. | Mesok. | Leptok. | V. Leptok. | Mesok. | Mesok. | Mesok. |
| sand [%] | 71 | 94 | 91 | 98 | 99 | 64 | 84 | 78 | 95 | 96 | 70 | 84 | 78 | 98 | 77 | 94 | 99 | 99 | 97 |
| mud [%] | 29 | 6 | 9 | 2 | 1 | 36 | 16 | 22 | 5 | 4 | 30 | 16 | 22 | 2 | 23 | 6 | 1 | 1 | 3 |

Table S2

| **Transect T8** | MIS 6 | | | | MIS 15 | | | | MIS 16 | | | | | MIS 17 | | | | |
| --- | --- | --- | --- | --- | --- | --- | --- | --- | --- | --- | --- | --- | --- | --- | --- | --- | --- | --- |
| lab No. | 18-1733 | 18-1734 | 18-1735 | 18-1736 | 18-1778 | 18-1779 | 18-1780 | 18-1782 | 18-1786 | 18-1787 | 18-1788 | 18-1789 | 18-1790 | 17-1791 | 17-1792 | 17-1793 | 17-1794 | 17-1795 |
| depths [cm] | 0-2 | 2-5 | 5-9 | 12-15 | 0-2 | 4.5-6 | 6-9 | 18-20 | 0-3 | 2-5 | 4-6 | 6-9 | 11-14 | 0-2 | 2-5.5 | 7-9 | 12-15 | 24-27 |
| description | top soil | tsunami | soil | dune | top soil | tsunami | soil | dune | top soil | pot. tsu | tsunami | soil | dune | top soil | pot. tsu | soil | dune | dune |
| short-chained [ng/g_TOC_] | 163562 | 40130 | 31095 | 24891 | 5106 | 79695 | 233733 | 331872 | 36380 | 16231 | 147087 | 84713 | 41714 | 105116 | 20755 | 38954 | 26380 | 105354 |
| long-chained  [ng/g_TOC_] | 483873 | 87895 | 45067 | 25843 | 13104 | 240577 | 903300 | 538846 | 129285 | 34472 | 383024 | 284928 | 59370 | 249169 | 29832 | 102376 | 59055 | 278112 |
| TAR | 15.6 | 8.3 | 10.8 | 6.0 | 12.5 | 8.9 | 20.6 | 14.8 | 23.9 | 10.7 | 13.8 | 22.0 | 7.5 | 21.7 | 11.2 | 21.7 | 12.1 | 11.7 |
| OEP | 4.9 | 2.9 | 8.1 | 3.6 | 3.7 | 1.6 | 6.4 | 5.6 | 0.9 | 1.4 | 2.2 | 3.7 | 4.4 | 3.3 | 4.6 | 4.2 | 6.2 | 5.0 |
| CPI_25-33_ | 5.3 | 4.1 | 11.5 | 3.1 | 4.6 | 3.0 | 8.0 | 6.3 | 3.7 | 2.1 | 3.0 | 4.2 | 5.7 | 3.4 | 3.4 | 4.6 | 2.3 | 6.3 |
|  |  |  |  |  |  |  |  |  |  |  |  |  |  |  |  |  |  |  |
| PAH 16 [ng/g_TOC_] | 834088 | 302352 | 4083 | 21403 | 2954 | 22163 | 78932 | 131819 | 13749 | 2578 | 28196 | 75095 | 11941 | 9046 | 1987 | 10464 | 27104 | 7123 |
|  |  |  |  |  |  |  |  |  |  |  |  |  |  |  |  |  |  |  |
| **Transect T3** | MIS 37 | | | | | MIS 38 | | | | MIS 39 | | | | MIS 40 | | | |  |
| lab No. | 18-1886 | 18-1887 | 18-1888 | 18-1889 | 18-1890 | 18-1891 | 18-1892 | 18-1893 | 18-1894 | 18-1895 | 18-1896 | 18-1897 | 18-1898 | 18-1899 | 18-1900 | 18-1901 | 18-1902 |  |
| depths [cm] | 1-4 | 5-8 | 8-10 | 11-14 | 18-24 | 2-4 | 4-7 | 7-10 | 14-18 | 0-4.5 | 6-8 | 9-12 | 15-18 | 0-2 | 4-5 | 6-9 | 31-36 |  |
| description | top soil | tsunami | soil | dune | dune | top soil | tsunami | soil | dune | top soil | tsunami | soil | dune | top soil | soil | dune | dune |  |
| short-chained [ng/g_TOC_] | 23110 | 395471 | 27453 | 141507 | 46615 | 51121 | 350524 | 11414 | 76267 | 25142 | 83031 | 83031 | 119518 | 1181496 | 53601 | 128352 | 98852 |  |
| long-chained  [ng/g_TOC_] | 163748 | 500160 | 62660 | 542698 | 53364 | 138499 | 487499 | 22206 | 190429 | 57325 | 144616 | 453386 | 1070634 | 3847704 | 126810 | 653304 | 281964 |  |
| TAR | 47.3 | 4.5 | 14.1 | 26.2 | 6.8 | 15.9 | 4.4 | 8.6 | 48.5 | 7.1 | 4.6 | 32.6 | 81.7 | 13.3 | 8.4 | 40.9 | 18.6 |  |
| OEP | 3.6 | 4.7 | 2.5 | 4.3 | 1.8 | 6.1 | 3.7 | 7.2 | 3.9 | 4.2 | 3.4 | 7.8 | 8.2 | 5.0 | 3.2 | 2.9 | 2.7 |  |
| CPI_25-33_ | 5.9 | 4.8 | 3.6 | 8.2 | 2.9 | 7.6 | 5.1 | 8.6 | 6.0 | 5.3 | 4.3 | 9.3 | 10.0 | 6.2 | 4.2 | 3.5 | 5.2 |  |
|  |  |  |  |  |  |  |  |  |  |  |  |  |  |  |  |  |  |  |
| PAH 16 [ng/g_TOC_] | 60165 | 2369428 | 33812 | 13060 | 118773 | 38367 | 490289 | 1033 | 31892 | 25079 | 302083 | 48459 | 80807 | 591692 | 98300 | 77168 | 61444 |  |

Table S3

| Name | MIS REF 2 | MIS REF 5 | MIS REF 6 | MIS REF 8 | MIS REF 9 | MIS REF 12 | MIS REF 13 | MIS REF 24 |
| --- | --- | --- | --- | --- | --- | --- | --- | --- |
| description | beach | soil | dune | beach | beach | beach | dune | beach |
| mean [Φ] | 1.83 | 1.34 | 1.63 | 0.62 | 0.42 | -0.12 | 0.90 | 1.34 |
| sortig [Φ] | 0.57 | 1.04 | 0.56 | 0.74 | 0.57 | 1.26 | 0.77 | 0.63 |
| skewness [Φ] | -0.18 | -0.11 | -0.03 | -0.11 | 0.10 | 0.21 | -0.05 | 0.02 |
| kurtosis [Φ] | 1.03 | 1.03 | 1.11 | 1.13 | 1.04 | 1.13 | 1.06 | 1.07 |
| mean | M. Sand | M. Sand | M. Sand | C. Sand | C. Sand | V. C. Sand | C. Sand | M. Sand |
| sortig | Moderately Well Sorted | Poorly Sorted | Moderately Well Sorted | Moderately Sorted | Moderately Well Sorted | Poorly Sorted | Moderately Sorted | Moderately Well Sorted |
| skewness | Coarse Skewed | Coarse Skewed | Symmetrical | Coarse Skewed | Fine Skewed | Fine Skewed | Symmetrical | Symmetrical |
| kurtosis | Mesokurtic | Mesokurtic | Leptokurtic | Leptokurtic | Mesokurtic | Leptokurtic | Mesokurtic | Mesokurtic |
| gravel [%] | 1.2% | 2.3% | 1.8% | 3.4% | 0.7% | 18.5% | 1.2% | 0.9% |
| sand [%] | 98.8% | 97.4% | 98.1% | 96.6% | 99.3% | 81.5% | 98.8% | 99.1% |
| mud [%] | 0% | 0.3% | 0.1% | 0% | 0% | 0% | 0% | 0% |
|  |  |  |  |  |  |  |  |  |

Table S4
